# Understanding variation in the clinical management of self-harm and suicidal ideation in hospital emergency departments: qualitative implementation study

**DOI:** 10.1192/bjb.2025.10161

**Published:** 2026-06

**Authors:** Selena O’Connell, Grace Cully, Sheena McHugh, Margaret Maxwell, Ella Arensman, Eve Griffin

**Affiliations:** 1 Postdoctoral Researcher, School of Public Health, University College Cork, Cork, Ireland; 2 Postdoctoral Researcher, National Suicide Research Foundation, Cork, Ireland; 3 Senior Postdoctoral Researcher, School of Public Health, University College Cork, Cork, Ireland; 4 Senior Postdoctoral Researcher, National Suicide Research Foundation, Cork, Ireland; 5 Senior Lecturer, School of Public Health, University College Cork, Cork, Ireland; 6 Professor of Health Services and Mental Health Research, Nursing, Midwifery and Allied Health Professions Research Unit, University of Stirling, UK; 7 Head of School, School of Public Health, University College Cork, Cork, Ireland; 8 Chief Scientist, National Suicide Research Foundation, Cork, Ireland; 9 Visiting Professor, Australian Institute for Suicide Research and Prevention, School of Applied Psychology, Griffith University, Brisbane, Australia; 10 Adjunct Professor, School of Public Health, University College Cork, Cork, Ireland; 11 Chief Executive Officer, National Suicide Research Foundation, Cork, Ireland

**Keywords:** Self-harm, suicide, emergency department, implementation, qualitative

## Abstract

**Aims and method:**

There is growing consensus on essential components of care for hospital-presenting self-harm and suicidal ideation, yet these are often inconsistently implemented. This qualitative study aimed to explore the implementation of components of care across hospitals. Interviews were conducted with health professionals providing care for self-harm and suicidal ideation in hospital emergency departments. Participants (*N* = 30) represented 15 hospitals and various professional roles. A framework analysis was used, where factors affecting each care component were mapped by hospital and hospital grouping.

**Results:**

A timely, compassionate response was facilitated by collaboration between liaison psychiatry and emergency-department staff and the availability of designated space. Other factors affecting the implementation of care components included patient preferences for, and staff encouragement of, family involvement, time taken to complete written care plans and handover and availability of next care impacting follow-up of patients.

**Clinical implications:**

The findings suggest a need for further integration of all clinical professionals on the liaison psychiatry team in implementing care for self-harm; improved systems of handover; further training and awareness on the benefits and optimal processes of family involvement; as well as enhanced access to aftercare.

Appropriate assessment and management of self-harm and suicidal ideation are key concerns for the field of liaison psychiatry, given the large number of these presentations to emergency departments.^1,2^ National Institute for Health and Care Excellence (NICE) guidelines on self-harm in the UK and the National Clinical Programme for Self-Harm and Suicide-Related Ideation (NCPSHI) in Ireland specify the key components of care that should be provided to those presenting with self-harm and suicidal ideation.^3,4^ These include a timely and compassionate response to the person presenting to the emergency department and biopsychosocial assessment by a mental health professional, involving family/carers, safety planning and follow-up. Although many of these components have been linked with reduced repetition of suicide and self-harm behaviours,^5–7^ studies have also highlighted variation and gaps in their implementation.^8–11^ A limited but growing body of literature has examined the factors contributing to variations in implementation, with the availability of dedicated resources found to be influential.^9,12^ A recent study of implementation of a national programme for self-harm in Ireland was shown to be effective, particularly in hospitals with no pre-existing services.^12,13^ The aim of the current study was to explore the implementation of key components of care for self-harm and suicidal ideation in Irish hospital emergency departments, and to understand the factors perceived by healthcare staff as contributing to these components of care, and to patient care pathways.

## Method

### Study design

Data were drawn from a larger study examining the impact of the national clinical programme NCPSHI.^12,14^ A qualitative study design was used to understand factors affecting implementation of the programme and its components, from the perspectives of providers and managers involved in its delivery.

### Setting and sample

Participants were recruited from 15 hospitals, which were divided into 3 groups based on their pre-existing services: designated liaison psychiatry service (group 1), liaison nurse service (group 2) and no designated staff (group 3). These hospital groups differed in regard to characteristics such as the annual number of self-harm attendances, staff availability to deliver the programme and observed changes in care outcomes post-implementation (Table 1).^12^

**Table 1:** Summary of hospital group features and implementation outcomes (adapted from Cully et al^12^)

Hospital data	Group 1 (7 hospitals)	Group 2 (4 hospitals)	Group 3 (4 hospitals)
Hospital information			
Pre-existing service/staff for self-harm	Designated liaison psychiatry service	Liaison nurse(s)	No designated staff
Self-harm attendances per year, mean	530	236	241
Details of early implementation of NCPSHI			
New staff appointments^a^	1.2	0.8	1.5
CNS cover, hours per day/days per week^b^	12+/7 service	8–9/5 service	12+/7 service
Out-of-hours cover in place	7 (100%)	1 (25%)	4 (100%)
Poisson regression model of pre- to post-implementation outcome changes, adjusted IRR (95% CI)			

Non-assessment	1.00 (0.94–1.06)	1.21 (1.10–1.33)^c^	0.78 (0.71–0.87)^c^
Mental health referral	0.99 (0.94–1.03)	1.15 (0.98–1.35)	1.39 (1.26–1.53)^c^
Medical admission	1.05 (0.99–1.12)	0.92 (0.83–0.97)^c^	0.91 (0.83–1.00)^c^
Psychiatric admission	0.99 (0.90–1.10)	0.85 (0.69–1.03)	0.92 (0.82–1.03)
Self-discharge	0.85 (0.79–0.92)^c^	1.06 (0.89–1.24)	1.01 (0.87–1.16)

NCPSHI,National Clinical Programme for Self-Harm and Suicide-Related Ideation; CNS, clinical nurse specialist; IRR, incidence rate ratio.

a. Number of new staff appointments (full-time equivalent), average number per hospital.

b. Reflects hours of cover for the majority of hospitals in given group.

c. Significant differences from pre- to post-implementation.

Sixty-five staff (previous or current) with at least 6 months’ experience in their role relating to the NCPSHI were invited to participate via email. Purposeful sampling was used to ensure equal representation from the hospital groups identified^12^ and relevant healthcare roles, including clinical nurse specialists (CNSs) and consultant psychiatrists responsible for delivering the NCPSHI and other relevant professionals, with an emphasis on those with experience of early implementation of the programme.

In total, 30 participants were recruited, comprising CNSs (*n* = 16) and consultant psychiatrists (*n* = 6) who delivered the NCPSHI, emergency medicine representatives (*n* = 2), nursing management (*n*= 2) and members of the national programme team (*n* = 4). Each of the three hospital groups was represented by participants in the study (Table 2). Participants had a median of 7.5 years of experience in their role on the programme. All participants provided written informed consent prior to participation.

**Table 2:** Sample characteristics

Role in relation to the programme *n* (%)	
Clinical nurse specialist	16 (53.3)
Consultant psychiatrist	6 (20.0)
National programme team member	4 (13.3)
Emergency medicine representative	2 (6.7)
Nursing management representative	2 (6.7)
Hospital group, *n* (%)	
Group 1	14 (53.8)
Group 2	4 (15.4)
Group 3	8 (30.8)
Years in role related to programme (median, interquartile range)	7.5 (6.0)

### Data collection

Individual semi-structured interviews were carried out. The topic guides were informed by the Consolidated Framework for Implementation Research 2.0 (CFIR).^15,16^ The topic guide included questions on perceptions and experiences of implementing each component of care, tailored to the professional’s role in implementation, and questions on the barriers and facilitators to implementation.^13^

Two researchers (S.O.C. and G.C.) conducted the interviews between November 2022 and April 2023. The interviews were predominantly conducted via video call (*n* = 27), with 3 conducted in person. The median length of interviews was 48 min (interquartile range 19). Interviews were audio-recorded and transcribed, and transcripts were pseudonymised.

### Data analysis

We examined the factors affecting implementation of each component of care across hospitals and hospital groups, drawing on the framework method.^17^ Data related to each care component, as well as barriers and facilitators of implementation, were coded as part of our previous study.^13^ NVivo (version 1.7 for Windows; Lumivero, Denver, CO, USA; https://lumivero.com/products/nvivo/) text-searching tools were used to identify terms relevant to care components, to ensure comprehensive coding of each. The primary author (S.O.C.) extracted information on experiences of implementing each care component, along with factors affecting implementation from NVivo to a matrix for each hospital. A summary of the implementation of each care component by hospital group was developed by S.O.C. based on reviewing and comparing experiences of hospitals within that group. Following this, an overall narrative was developed to summarise key factors affecting the implementation of each care component within the overall sample, and patterns across hospital groups. In order to enhance the credibility and confirmability of the findings, coded data and text summaries were checked by G.C. for a sample of care components (timely and compassionate response, biopsychosocial assessment and emergency care plan) across each hospital group. Limited discrepancies were identified, and the primary author (S.O.C.) completed double-checking of data extraction and summary for the remaining components. The findings were reviewed by the research team and presented to NCPSHI staff for feedback.

## Results

**Fig. 1:**
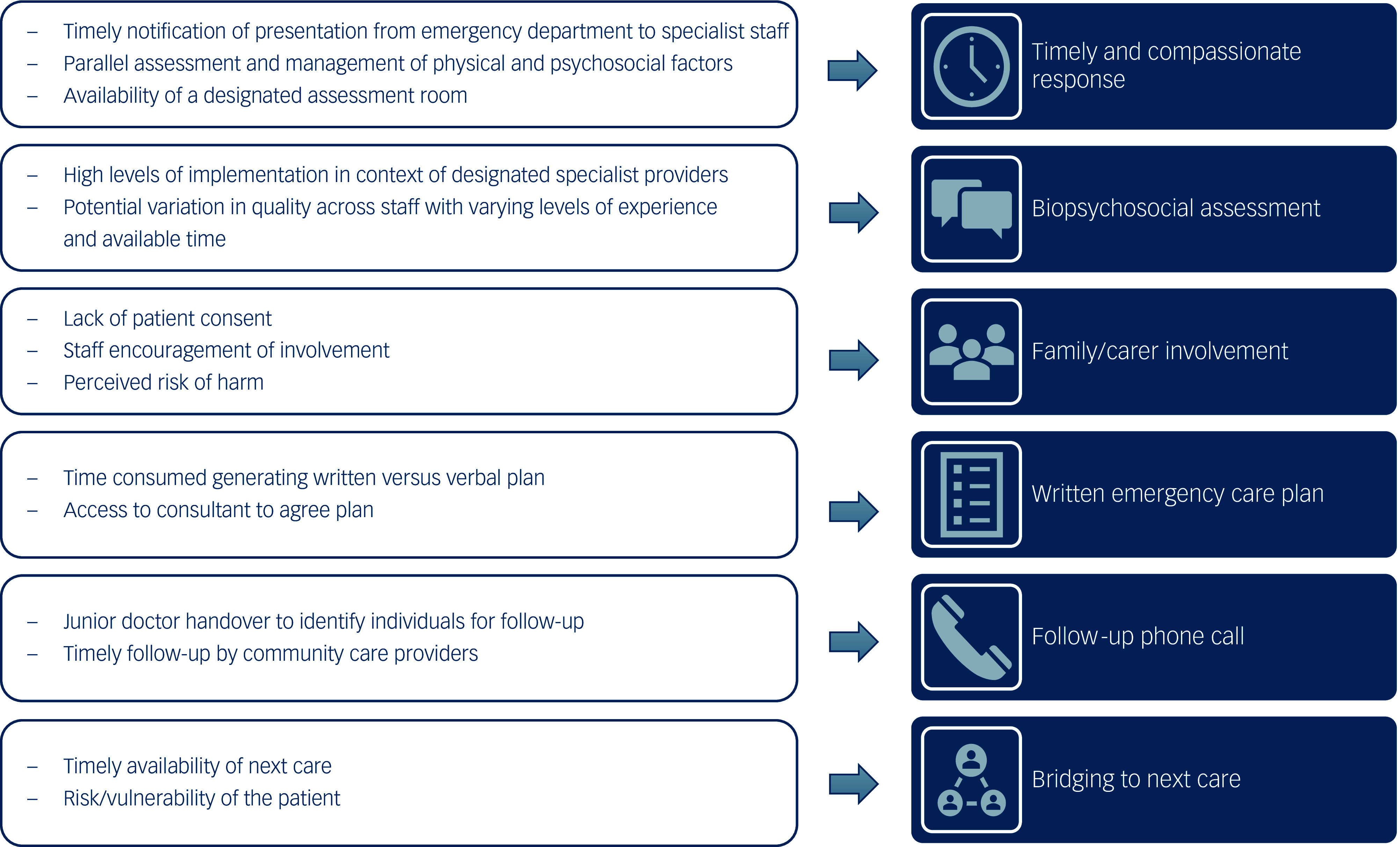
Factors affecting implementation of components of the care pathway.

Factors were identified that uniquely influenced the implementation of each key component of care (Fig. 1). Most barriers and facilitators were common across hospital groups. However, groups varied in regard to their delivery of a timely response, with hospitals having pre-existing liaison psychiatry services (group 1) describing fewer challenges in delivering this care component.

### A timely and compassionate response

From the point of entering the emergency department, there was variation in participants’ description of the timeliness of care. Participants’ accounts varied in regard to the time taken to notify programme CNSs of a relevant presentation following triage. Generally, early communication was perceived as beneficial and was most commonly reported in hospitals with pre-existing liaison psychiatry services (group 1), with the aid of designated protocols within the emergency department and established relationships with emergency-department staff. The timeliness of biopsychosocial assessments was influenced by the availability of a designated assessment room. Hospitals with pre-existing liaison psychiatry services (group 1) tended to have greater access to designated rooms compared with other groups, with this improving across groups over time:

‘We have an incredibly positive working relationship with the liaison service, which is definitely the primary driver of parallel assessment, because there’s that trust and understanding by the nature of working together on a floor on a daily basis, whereby we know that we can trust the liaison team to do what they need to do to progress an assessment. They know that we can do what we need to do, such that nothing is missed medically.’ (Emergency medicine consultant, group 1).

The NCPSHI recommended parallel assessment and management of physical conditions alongside psychosocial factors. While early notification was positive in that it enabled CNSs to engage with the person early and reduce the risk of patients leaving the emergency department without assessment, CNSs reported that early referral to the liaison team could be problematic when emergency-department staff viewed the liaison team as ‘taking over the patients’ care’; CNSs also reported difficulties where there remained unresolved medical issues, or medical symptoms that subsequently emerged. This barrier was reported to some extent across all the hospital groups, and participants reported that liaison psychiatry and emergency-department teams in many hospitals were collaborating to improve these processes.

### Biopsychosocial assessment

This assessment was seen as a standard and valuable practice by all participants:

‘It gives you a good ground for your practice and your intervention with the patient and, in itself, it can be very therapeutic. Often, patients would report feeling listened to and felt much better having gone through the initial assessment process.’ (CNS, group 1).

Most hospitals with pre-existing services (groups 1 and 2) were already conducting biopsychosocial assessments before introduction of the programme, and so it did not represent a substantial change to their practice. Hospitals with no pre-existing services (group 3) had no CNSs providing assessment prior to the programme.

While assessments were conducted using proformas that enhanced consistency, some participants reported that quality of assessment could be affected at night when conducted under time pressures, primarily by junior doctors providing out-of-hours cover. To counter this, staff in some hospitals provided training on conducting assessments to junior doctors as part of their induction, and some created packages for junior doctors that included a proforma for the assessment as well as programme documents necessary for implementation.

### Family/carer involvement

Participants emphasised that they tried to involve family/carers because it was recommended by the programme and seen as beneficial to building a picture of the patient and developing an appropriate care plan. A common challenge across hospitals was lack of patient consent for family involvement. Some participants described how they would gently encourage patients to involve family/carers and describe the rationale for their involvement. This encouragement was a particularly strong feature of participants’ accounts in hospitals with no pre-existing services (group 3):

‘So I would explain to them, it’s for their support, and I would say, “You’ve come here for help, and this is one way of helping you, if you’re feeling suicidal or self-harming again.” And to be honest, it worked the majority of the time […] and they would change their mind, and say okay. And they would give me contact details of somebody. Now it might be a friend; it was somebody that they felt comfortable in confiding in.’ (CNS, group 3).

However, if a patient did not consent to family involvement, participants spoke of balancing confidentiality with the risks of adverse outcome such that confidentiality would be broken if the risk of adverse outcome was deemed high:

‘It can be difficult to involve the families and carers. However, if the risk was so significant, we could involve families and carers even without the consent of the individual. Because the safety is a higher priority than the confidentiality.’ (CNS, group 2).

There were also reports of practical barriers to the involvement of family/carers, such as difficulties reaching family, particularly at night, or families being unhelpful in the process. Staff highlighted that collateral information could be sought from GPs as an alternative when family/carers could not be involved.

### Written emergency care plans

Written emergency plans (ECPs) were commonly implemented, but there was variation in the consistency and fidelity of their implementation according to participants. Participants across hospital groups highlighted the benefits of ECPs as something that a patient and their family could look at after leaving the emergency department:

‘I find the emergency care plans for us, well, personally speaking anyway, but I find them really good because you’re making sure that the person understands what they’re going home with. You’re making sure they understand their follow-up. […] It nearly felt like another part of a safety planning and safety netting, that they have something to go home with and to refer to.’ (CNS, group 3).

However, ECPs were typically handwritten and considered time-consuming, which hindered their implementation. Some participants described cases where patients left with a verbal ECP due to eagerness to leave and the time taken to complete the written plan. In addition, the timeliness of consultant input to agreeing the care plan was noted to vary across groups. For hospitals without designated consultant psychiatrists to support the programme in the early years, CNSs reported delays in accessing a consultant to agree to the plan before returning to the patient with the final plan and recommendations. There were also different templates used for ECPs, and some participants highlighted the need for greater standardisation of the ECP.

### Twenty-four hour follow-up phone call

The NCPSHI specified the need for telephone follow-up of the patient within 24 h of leaving the emergency department. This follow-up was mostly a new practice in those hospitals without pre-existing designated liaison psychiatry services. There were no clear hospital group differences in participants’ experiences of conducting the follow-up call. Participants across hospital groups emphasised the benefits of the follow-up call, how it was appreciated by patients, was an opportunity to reinforce the care plan and provided some reassurance to staff:

‘And that was very reassuring for us because that meant that the person is post-acute, it’s a day later, it’s a follow up for starters, it’s a follow-up to check-in that they know what the plan is and that they can have further signposting.’ (Consultant psychiatrist, group 1).

CNSs reported conducting the follow-up call in most cases, except where a person was referred to or previously linked in with specialist next care, most often a community mental health team, where contact was made with that service instead. CNSs would often still contact the patient directly, in particular at weekends, when the community team would not be available, or where there were extended waiting times for next care:

‘If the patient was referred to the community team, that follow-up wouldn’t be done because the community team would do it. The biggest problem arose if a patient presented on a Friday, who was the follow-up on the Friday and the Saturday […]. So as a team then we […] have a book which we leave people for follow-up calls for the weekend.’ (CNS, group 1).

There was a perceived risk that a follow-up call would not be implemented for patients seen during the night by junior doctors. Participants reported issues with handover, which meant that some patients had been missed for follow-up, and continually sought to clarify and improve handover systems with junior doctors:

‘The follow-up, what has worked well is where the clinical nurse specialist works closely with the [junior doctors] and ensures that they get good handover in the morning, that everybody knows what’s going on.’ (National programme team member).

### Bridging to next care

Within the NCPSHI, bridging to next care involves continued engagement until a patient reaches their next care appointment. Participants indicated that bridging was often difficult to implement, due to extensive waiting times for next care within some catchments and the resulting high workload in maintaining extended bridging over several weeks. This increased workload deterred bridging altogether in some sites because it was seen as unrealistic in the context of other role demands. Participants often described operating a ‘case-by-case basis’, and prioritised patients who were seen as vulnerable or warranting urgent next care:

‘If you are actually bridging, it could go on for a week, it could go on for two weeks, it could go on for four weeks. So you’re … you could be phoning the person twice a week or three times a week and you’re keeping records. That means then … so that, straightaway, is adding to the work. […] Now, what I would say, [researcher], is that […] we do decide based on the individual.’ (CNS, group 1).

Participants also highlighted the challenge of people who frequently re-attend the emergency department. While re-attendance may be appropriate in some cases, there was a concern among some participants that certain patients’ needs may be more appropriately met in community or primary care settings. To address this, participants reported working with next-care providers to agree a care plan, and some participants have provided extended models of follow-up such as out-patient visits, or suggested these as a way to reduce re-attendance at the emergency department.

## Discussion

This study explores the implementation of a model of care for self-harm and suicidal ideation from the perspective of providers, and identifies factors influencing implementation. Our findings illuminate how a more timely patient journey can be facilitated through collaboration between liaison psychiatry and emergency medicine staff (see practice and policy implications in Table 3). The timeliness of the patient journey was also influenced by access to a designated assessment room. Our previous work^13^ has detailed strategic approaches to securing such space through collection of audit data and local hospital advocacy.

**Table 3:** Factors affecting implementation of care components and associated practice and policy implications

Care component	Implementation factors	Practice and policy implications
Timely and compassionate response	Timely notification of presentation from emergency department to specialist staff Parallel assessment and management of physical and psychosocial factors Availability of a designated assessment room	Support hospitals introducing new specialist providers/liaison psychiatry roles to develop collaboration with emergency-department staff Advocate for designated assessment room (providers) Data on assessment room requirements (strategic)
Biopsychosocial assessment	High levels of implementation in context of designated specialist providers Potential variation in quality across staff with varying levels of experience and available time	Encourage training opportunities for the range of implementation providers, including junior doctors Make biopsychosocial assessment proformas readily available
Family/carer involvement	Lack of patient consent Staff encouragement of involvement Perceived risk of harm (outweighing lack of consent)	Clarify benefits of family involvement to patient Further clarify protocol when patients do not consent to involvement
Written emergency care plan	Time consumed generating written versus verbal plan Access to consultant to agree plan	Proformas for emergency plan Ensure designated consultant available to support clinical nurse specialists Strengthen evidence base and communication of findings
Follow-up phone call	Junior doctor handover to identify individuals for follow-up Timely follow-up by community care providers	Strengthen handover systems between junior doctors and CNSs providing follow-up
Bridging to next care	Timely availability of next care Risk/vulnerability of the patient	Develop collaboration with community providers to agree plans Increase resource and capacity of community providers to provide quicker service access

The study findings highlight the role of the entire liaison psychiatry team in providing the key components of care. Healthcare professionals in this study reported that biopsychosocial assessment was consistently implemented, corroborating previously observed improvements in rates of non-assessment for hospitals without pre-existing services,^12^ and highlighting the positive impact of introducing dedicated CNSs and the need for a dedicated consultant to be available from the outset of delivering such components of care. Additionally, the study highlights the role of junior doctors in delivering the programme out of hours, with some challenges in terms of consistent assessment and handover of cases. To reduce this gap, junior doctors should be integrated within the team-based approach and provided with regular training to support both assessment and follow-up – for example, through a train-the-trainer model as used for emergency-department staff. These findings also suggest the need to ensure sufficient handover systems and availability of assessment proformas.

This study echoes previous findings of mixed perspectives on the advantages and disadvantages of family involvement.^18,19^ A previous review suggested that this may be facilitated by the clinician directly inviting family involvement, exploring fears and understanding preferences.^18^ Staff in this study also described ways in which they facilitate involvement through explaining the benefits of family involvement, although these accounts were not provided by all staff. Additional training and awareness in this may help with implementation. However, there is also a need for further research to clarify best practice in involving family/carers.

Healthcare professionals in this study indicated that the time taken to complete written ECPs acted as a barrier to their implementation. This may also reflect other challenges such as readiness to integrate ECPs as a new practice and/or the ECP being perceived as a low priority, a pattern seen in previous research.^20,21^ To inform future implementation of ECPs, it is important to strengthen the evidence base around safety planning and the conditions under which it is effective, and to communicate these findings to practitioners.

In terms of aftercare, challenges were noted in bridging to next care given the lack of consistency in the timely availability of next care. Lack of aftercare may contribute to increased repetition of self-harm and re-presentation to the emergency department,^22^ as well as to patients feeling isolated in their care.^23^ Therefore, enhancing access to aftercare is a priority for service development, via greater collaboration and integration between hospital and community/primary care providers.^24^ Quinlivan and colleagues^24^ also identified the potential to provide psychological interventions as part of liaison psychiatry services, which was also proposed by some participants in our study. The optimal models of providing psychological therapy through liaison services require further examination.

### Strengths and limitations

This qualitative study used an implementation framework to explore factors affecting the care pathway of people presenting to an emergency department with self-harm or suicidal ideation, demonstrating the primary factors affecting delivery of each care component and offering explanations for the variation observed in a previous impact study.^12^ However, it was not possible to directly compare and attribute experiences of implementation with implementation outcomes at a hospital level. A strength of this study is the inclusion of a range of professionals involved in the delivery of care. However, this study is limited in that it does not include the perspectives of people attending the emergency department or their families. Given the findings of varying experiences of compassion from a patient perspective and the impact of this on patient experience,^25^ it would be important to further examine the concept of compassion and how it is embodied within these interactions. Some research is under way in this area – for example, using conversation analysis of emergency-department psychosocial assessments.^26^

### The care pathway

The care pathway for people presenting to emergency department with self-harm or suicidal ideation is subject to variation, despite recommendations for best practice. This study identifies factors influencing the implementation of care components and draws out practical and clinical implications to address current barriers to implementation, including supporting collaboration between liaison psychiatry and emergency medicine professionals and further integration of all clinical professionals within liaison psychiatry. Evidence-based training on family involvement and safety planning may help address current challenges, as well as further research on optimal systems of follow-up.

## Data Availability

Data cannot be made available due to difficulties involved in protecting the identity and confidentiality of participants drawn from a small population.

## About the authors

**Selena O’Connell** is a Postdoctoral Researcher at the National Suicide Research Foundation and School of Public Health, University College Cork, Ireland. **Grace Cully** is a Senior Postdoctoral Researcher at the National Suicide Research Foundation and School of Public Health, University College Cork, Ireland. **Sheena McHugh** is a Senior Lecturer at the School of Public Health, University College Cork, Ireland. **Margaret Maxwell** is Professor of Health Services and Mental Health Research in the Nursing, Midwifery and Allied Health Professions Research Unit, University of Stirling, UK. **Ella Arensman** is Professor of Public Mental Health at the School of Public Health, University College Cork, Ireland and Visiting Professor at the Australian Institute for Suicide Research and Prevention, Griffith University, Australia. **Eve Griffin** is Chief Executive Officer of the National Suicide Research Foundation, Cork, Ireland and Adjunct Professor at the School of Public Health, University College Cork, Ireland.
